# pH‐Regulated Synthesis of Multi‐Shelled Manganese Oxide Hollow Microspheres as Supercapacitor Electrodes Using Carbonaceous Microspheres as Templates

**DOI:** 10.1002/advs.201400011

**Published:** 2014-12-10

**Authors:** Jiangyan Wang, Hongjie Tang, Hao Ren, Ranbo Yu, Jian Qi, Dan Mao, Huijun Zhao, Dan Wang

**Affiliations:** ^1^ State Key Laboratory of Multiphase Complex SystemsInstitute of Process Engineering Chinese Academy of Sciences No. 1 Beiertiao, ZhongguancunBeijing100190P. R. China; ^2^ Department of Physical Chemistry, School of Metallurgical and Ecological EngineeringUniversity of Science and Technology BeijingNo. 30, Xueyuan RoadHaidian District Beijing100083P. R. China; ^3^ Centre for Clean Environment and EnergyGold Coast Campus Griffith UniversityQueensland4222Australia; ^4^ University of Chinese Academy of SciencesNo.19A Yuquan RoadBeijing100049P. R. China

**Keywords:** pH‐regulated, hard‐templated method, multi‐shelled hollow microsphere, manganese oxide, supercapacitor

## Abstract

**Multi‐shelled Mn_2_O_3_ hollow microspheres** have been achieved through a pH‐regulated method and used as supercapacitor electrodes. The designed unique architecture allows efficient use of pseudo‐capacitive Mn_2_O_3_ nanomaterials for charge storage with facilitated transport for both ions and electrons, rendering them high specific capacitance, good rate capability, and remarkable cycling performance.

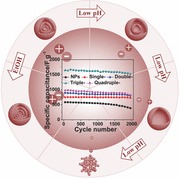

Supercapacitors are intermediate devices between conventional batteries and dielectric capacitors that can be used in various electric systems.^1^ The pseudo‐capacitors have drawn great attentions because of their intrinsic properties of fast and reversible surface redox reactions that enable high charge storage capacities greater than double‐layer supercapacitors. Besides, a main attraction of pseudo‐capacitors is their ability to embrace the advantages of both lithium ion batteries (LIBs) (high energy density) and capacitors (high power density).^2^ Different transition metal oxides,[[qv: 3a,b]] such as RuO_2_,[[qv: 3c]] CoO_x_,[[qv: 3d]] NiO,[[qv: 3e]] and MnO_x_,[[qv: 3f]] have been investigated as supercapacitor electrode materials. Among them, the manganese oxide is one of the most promising candidates due to its high specific capacitance, superior environmental compatibility and cost effectiveness.^4^ Manganese oxides can be in various oxidation states and chemical forms. The most studied manganese oxides include MnO_2_ (Mn[IV]),[[qv: 5a]] Mn_2_O_3_ (Mn[III])[[qv: 5b]] or Mn_3_O_4_ (Mn[II/III]).[[qv: 5c]] Though manganese oxides have demonstrated huge potentials as supercapacitor electrodes, they still suffer from some drawba­cks:[[qv: 2e]],[[qv: 2f]],[[qv: 4a]],^6^ 1) low specific surface area, resulting in less faradic active sites, hence lower specific capacitance; 2) poor electron and ion conductivities, unfavorable for high rate of charge/discharge, leading to a lower power density; 3) partial dissolution in the electrolytes during cycling, giving rise to the capacitance degradation.

An emerging solution to overcome the above drawbacks is to utilize the unique structural characteristics of the hollow micro‐/nano‐structured materials.^7^ The advantages of adopting hollow nanostructures as the supercapacitor electrodes are obvious and listed below:^8^ 1) the unique hollow structure provides more accessible faradic reactive sites in real capacitive process as a result of the enhanced surface‐to‐volume ratio and/or enlarged effective surface area per unit mass, leading to a higher energy density; 2) the porous shells can dramatically enhance the accessibility of the electrolyte to the active manganese oxide surface with improved conductivity and shortered transport length for both ions and charges and thus the rate capability and power density can be improved; 3) importantly, with the multi‐shelled hollow structures, different shells are supported each other and the exterior shell protects the interior shells from the electrochemical dissolution to achieve better structural and electrochemical stabilities, leading to an improved cycling performance. However, to allow us to take full advantage of multi‐shelled hollow spheric structures, an enabling synthetic method must be developed to achieve precise control of key structural parameters such as the thickness, particle size, pore size and porosity of shells, and the number, diameter and spacial arrangement of shells.

Recently, a general method utilizing the carbonaceous microspheres (CMSs) as sacrificial templates has been developed by our group for controllable synthesis of hierarchical multi‐shelled hollow microspheres with adjustable nanoparticle subunits and shell structures.[[qv: 8a]],^9^,^10^ The method has been successfully applied for synthesis of a variety of multi‐shelled metal oxide hollow microspheres (e.g., ZnO,[[qv: 10b]] Co_3_O_4_,[[qv: 9a]] Fe_2_O_3_,[[qv: 9b]] SnO_2_[[qv: 10c]] and MFe_2_O_4_[[qv: 10a]] (M = Zn, Ti, etc.)) with elaborate control over the number of internal shells, shell spacing, and exterior shell structure. To date, however, the multi‐shelled hollow structured MnO_x_ materials have not been used for supercapacitor applications mainly due to the difficulties in using the template methods to achieve the control of composition, crystalline phase and microstructure (such as shell number, shell thickness, porosity) of MnO_x_.[[qv: 2a]],[[qv: 3a,b]],[[qv: 4a]]

Herein, we report an effective approach to synthesize the multi‐shelled Mn_2_O_3_ hollow microspheres with controlled number of shells, shell thickness and porosity as the high performance supercapacitor electrode materials. The as‐synthesized triple‐shelled Mn_2_O_3_ hollow microspheres with thin porous shells show an extremely high specific capacitance up to 1651 F g^–1^ at a current density of 0.5 A g^–1^, and remarkable cycling stability with 92% retention after 2000 consecutive cycles. Besides, the rate capability is also impressive, showing a specific capacitance as high as 1422 F g^–1^ at a high current density of 10 A g^–1^. To our knowledge, the performance of Mn_2_O_3_‐based electrode materials for supercapacitor reported here is a new record to date.

As previously reported, the control over the crystalline phase and microstructure (including the shell thickness, shell number and porosity, etc.) can be achieved by tuning the metal ions adsorption amount and the depth embedded into the carbonaceous microsphere templates, as well as tuning the annealing conditions.[[qv: 8a]],^9^,[[qv: 10a]] By simply increasing the precursor salt concentration, more metal ions can penetrate deep into the CMSs templates, resulting in metal oxides with more shells after heat treatment. However, simultaneously, the excess metal ions are more inclined to rapidly accumulate on the surface of CMSs rather than the inner parts of CMSs, lowering the uniformity of hollow spheres and increasing the particle size and thickness of shells, unfavourable for high performance supercapacitors. Thus, it is quite challenging to control such structural variables, especially the nanoparticle size and shell thickness, by simply controlling the salt concentration as we previously did.

In this communication, we report a modified control synthesis approach for fabrication of uniform single‐, double‐, triple‐shelled hollow microspheres with thin porous shells by precisely controlling the pH values of the precursor solutions with an appropriate concentration. As shown in **Figure** **1**, by simply adjusting the pH of 1.0 M Mn(Ac)_2_ precursor solution at 3.35, 4.43 and 6.43, respectively, the single‐, double‐ and triple‐shelled hollow microspheres can be obtained in a high yield after the removal of the templates by heat treatment (see experiment details in Table S1, Supporting Information). It is well known that a decrease in pH leads to a decrease in the surface negative charge of CMSs, hence a decreased Mn cations adsorption onto the CMSs template.[[qv: 10c]] As shown in Figure S1, Supporting Information, the zeta potential of carbonaceous microsphere templates in aqueous solution is about –44.5 mV at the pH value of 6.78. With the solution pH values of 3.52, 4.80 and 6.23, the corresponding zeta potentials of CMSs are found to be –20.4, –29.9, and –42.0 mV, respectively, meaning a less electrostatic attraction between the negatively charged CMSs and the Mn cations. Moreover, with the addition of HCl aqueous solution (pH regulator), the coordination group of Mn^2+^ ions are changed from [Mn(H_2_O)_6_]^2+^ to [Mn(H_2_O)_6‐x_Cl_x_]^2‐x^ (0 < *x* < 2, when 0 ≤ pH ≤ 7), further decreasing the electrostatic attraction.^11^ As the adsorbed Mn ions by CMSs decrease, Mn_2_O_3_ hollow microspheres with certain number of shells can be obtained after heat treatment (Figure S2, Supporting Information, **Figure** **2**a,b,d,e). It should be noted that when the solution pH is below 1, the CMSs could only take very limited amount of Mn ions, leading to the formation of Mn_2_O_3_ nanoparticles (NPs) instead of Mn_2_O_3_ hollow microspheres (Figure S3, Supporting Information).

**Figure 1:**
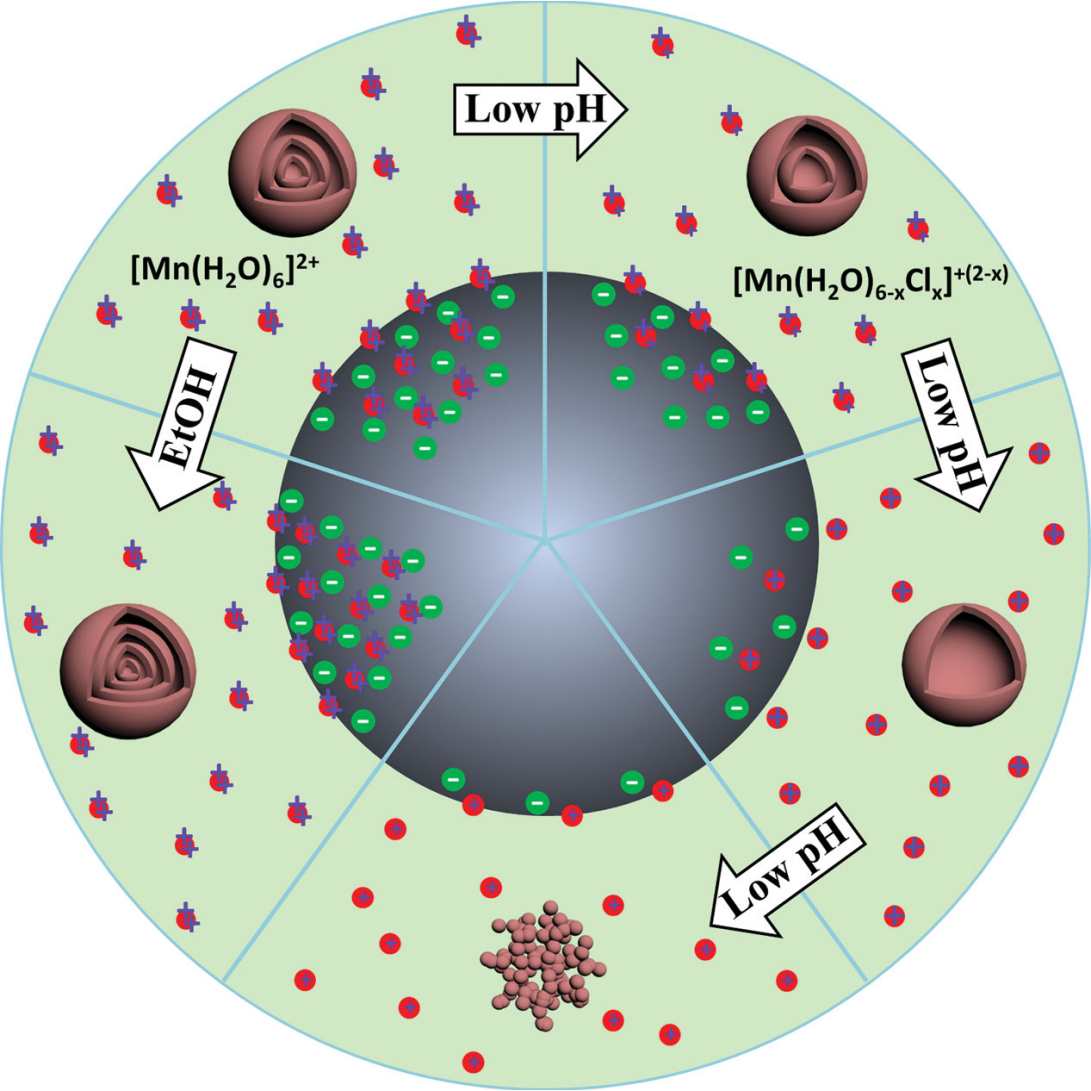
Scheme of synthesis mechanism for Mn_2_O_3_ nanoparticles and multi‐shelled hollow microspheres under different adsorption conditions.

**Figure 2:**
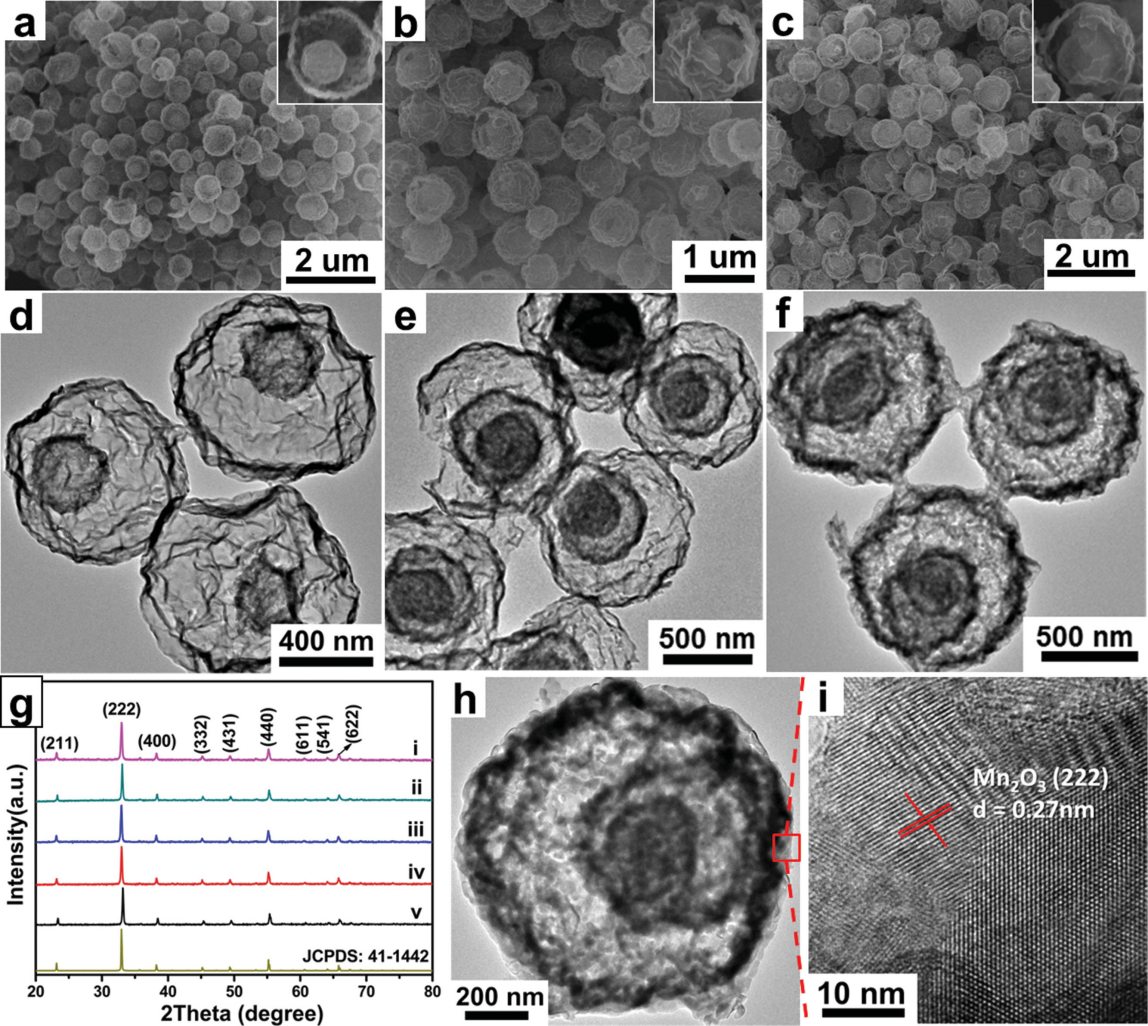
a,b,c) SEM and d,e,f) TEM images of double‐, triple‐, quadruple‐shelled Mn_2_O_3_ hollow microspheres. g) XRD patterns of as‐prepared Mn_2_O_3_ hollow microspheres and Mn_2_O_3_ nanoparticles (i, ii, iii, iv, and v represent single‐, double‐, triple‐, quadruple‐shelled Mn_2_O_3_ hollow microspheres and Mn_2_O_3_ nanoparticles, respectively). h,i) TEM and HRTEM images of an individual quadruple‐shelled Mn_2_O_3_ hollow microspheres.

Besides exploiting the pH effect, we can further strengthen the adsorption of Mn ions by raising the adsorption temperature to 30 °C and adding appropriate concentration of ethanol (EtOH) into the aqueous infusion solution (water : EtOH = 1:3, v/v). According to the Arrhenius equation,[[qv: 9a]],^12^ a higher temperature value results in a higher diffusion rate coefficient, and thus more Mn ions can be absorbed by CMSs readily. Moreover, ethanol can improve the surface wettability of CMSs and thus facilitate the Mn ion adsorption deep into the CMSs templates.[[qv: 9b]],^13^ As a result, quadruple‐shelled Mn_2_O_3_ hollow microspheres can be obtained (Figure 2c, 2f).

The crystalline structures of as‐prepared Mn_2_O_3_ hollow microspheres and nanoparticles were investigated by X‐ray diffraction (XRD). As shown in Figure 2g, all reflection peaks can be indexed to a pure Mn_2_O_3_ phase (JCPDS card no. 41–1442), with no additional peaks are detected.^14^ It is worthy to note that there are not any other types of MnO_x_ that exist in the resulting products, such as MnO_2_, probably due to there not being enough driving force to step over the energy barrier to a much higher valence state. Further compositional information can be obtained by Raman spectra (Figure S4, Supporting Information), EDX and corresponding elemental mapping (Figure S5, Supporting Information). The average grain sizes of Mn_2_O_3_ nanoparticles and hollow microspheres with single‐, double‐, triple‐, and quadruple‐shells calculated by Scherrer Equation are 19.54, 30.35, 28.43, 27.12 and 29.87 nm respectively, consistent with the TEM (transmission electron microscopy) and SEM (scanning electron microscopy) observations shown in Figure 2, S2 and S3, Supporting Information. Moreover, the TGA‐DTA (thermo gravimetric analysis – differential thermal analysis) data (Figure S6, Supporting Information) reveal that the weight loss stopped at ≈325 °C. According to the actual heat treatment conditions (500 °C, 1 h), we can anticipate that the obtained multi‐shelled hollow microspheres are made of pure Mn_2_O_3_ phase with high crystallinity, which is also proved by the high‐resolution TEM (HRTEM) image (Figure 2i) with commonly observed (222) lattices. X‐ray photoelectron spectroscopy (XPS) was utilized to understand the chemical composition of the as‐prepared multi‐shelled Mn_2_O_3_ (Figure S7). Taking triple‐shelled Mn_2_O_3_ hollow microspheres as a representative example, two characteristic broad peaks encompassing the Mn (III) 2p_3/2_ at 641.8 eV and 2p_1/2_ at 653.3 eV are observed, with a spin‐energy separation of 11.5 eV, which is consistent with the reported data of Mn (III) 2p_3/2_ and Mn (III) 2p_1/2_ in pure Mn_2_O_3_ phase.^15^

The performance of the synthesized multi‐shelled Mn_2_O_3_ hollow microspheres as electrode materials for supercapacitors were evaluated by a three‐electrode electrochemical system.^16^ **Figure** **3**a shows the cyclic voltammetric (CV) curves of the triple‐shelled Mn_2_O_3_ hollow microspheres electrodes obtained from 6 m KOH electrolyte under scan rates ranged from 5 mV s^–1^ to 200 mV s^–1^ within the potential window of 0.0–0.45 V (vs. SCE (saturated calomel electrode)). For comparison, the electrodes assembled with single‐, double‐, quadruple‐shelled Mn_2_O_3_ hollow microspheres and Mn_2_O_3_ NPs are also evaluated (Figure S8, Supporting Information). It can be found that the CV curves of Mn_2_O_3_ are distorted from mirror image symmetry and a pair of redox peaks can be clearly observed, which can be ascribed to the redox reactions between the oxidation states of Mn(III) and Mn(IV). Therefore it can be known the Faradic capacitance plays the main role in the charge storage of Mn_2_O_3_ hollow structures. Moreover, all these CV curves preserve the obvious redox peaks of Mn_2_O_3_ even at a high scan rate of 200 mV s^–1^, indicating an ideal capacitive behavior and desirable fast charge/discharge property for power devices.

**Figure 3:**
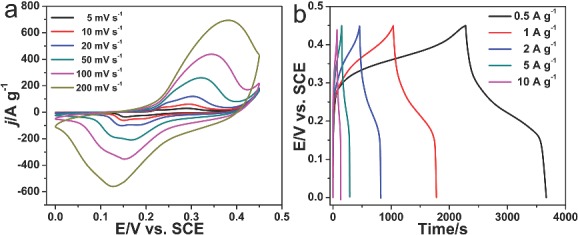
a) CV curves of triple‐shelled Mn_2_O_3_ hollow microspheres at various scan rates of 5, 10, 20, 50, 100, 200 mV s^–1^ . b) Galvanostatic charge/discharge curves of triple‐shelled Mn_2_O_3_ hollow microspheres at different current densities in the voltage range of 0–0.45 V.

To further evaluate the cell performance, the galvanostatic charge/discharge curves at various current densities were measured with a constant voltage window of 0.0–0.45 V.^17^ As shown in Figure 3b, the linear voltage‐time profiles reveal almost symmetric charge/discharge characteristics without obvious iR drop, indicating excellent capacitive characteristics of rapid *I*–*V* responses and small equivalent series resistance, ideal for capacitive device applications. The specific capacitance of the triple‐shelled Mn_2_O_3_ hollow microspheres electrode was found to decrease with increasing discharge current densities. It can be seen from Figure S9, Supporting Information, that the specific capacitance of triple‐shelled Mn_2_O_3_ hollow microspheres was 1651 F g^–1^ at a discharge current density of 0.5 A g^–1^ and still kept as high as 1422 F g^–1^ at a high discharge current density of 10 A g^–1^, indicating a superior rate performance. Remarkably, compared with other Mn_2_O_3_‐based supercapacitor electrode reported up to date, the specific capacitance of the triple‐shelled Mn_2_O_3_ hollow spheres is a new record (Table S2, Supporting Information).

The shell structures of the Mn_2_O_3_ hollow microspheres can be well controlled to optimize the capacitive performance. **Figure** **4**a compares the CV curves of Mn_2_O_3_ NPs and hollow microspheres with different shell numbers (1–4) to explore the effect of shell numbers on the resultant capacitances. It can be seen that the CV integration areas per weight of triple‐shelled Mn_2_O_3_ hollow microspheres, which indicates the charge stored in the supercapacitor, are much larger than that of Mn_2_O_3_ NPs, single‐, double‐ and quadruple‐shelled hollow microspheres. It seems that the maximum capacitance could only be obtained from a moderate shell number of Mn_2_O_3_ hollow microspheres with the porous thin shell. This is easy to be understood that as the maximal active sites and appropriate pore volume could only be obtained from the proper shell structures to facilitate the ions penetration and the process of redox reactions. The specific capacitance of the electrodes with different scan rates is given in Figure 4b. Specific capacitance of the electrode was calculated by integrating the voltammetric charge in the CV curves.^18^ At the same scan rate, the resulting specific capacitance for the multi‐shelled Mn_2_O_3_ hollow microspheres electrodes were much higher than that of the Mn_2_O_3_ NPs, and among them triple‐shelled Mn_2_O_3_ hollow microspheres achieved a maximum capacitance, the crest value of specific capacitance of 1640 F g^–1^ at the scan rate of 5 mV s^–1^ can be achieved with the triple‐shelled Mn_2_O_3_ hollow microspheres. The same trend can also be observed by comparison of galvanostatic charge/discharge curves of various samples at the same charge/discharge current density (Figure 4c).

**Figure 4:**
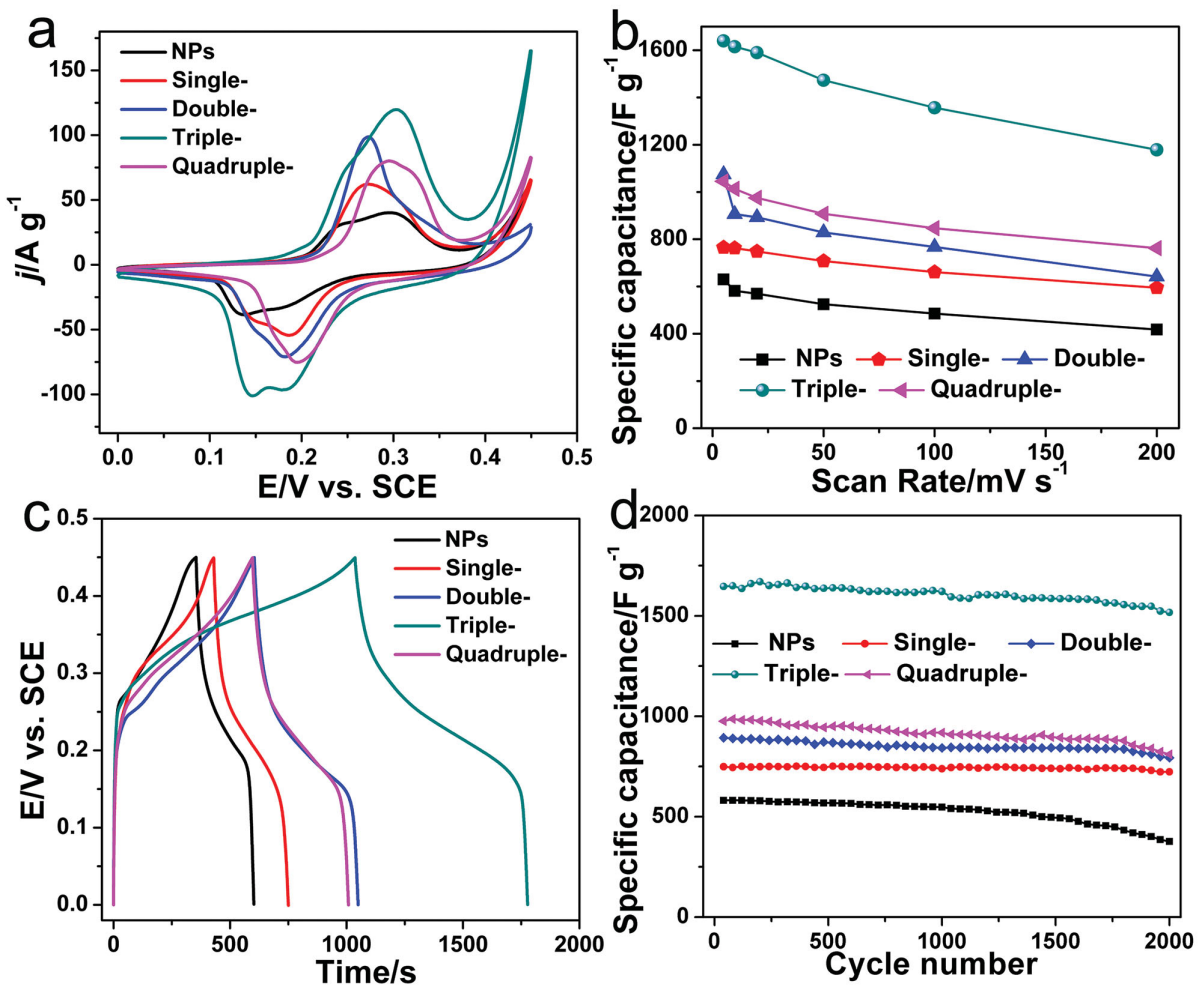
a) Comparison of CV curves of Mn_2_O_3_ nanoparticles, single‐, double‐, triple‐ and quadruple‐shelled Mn_2_O_3_ hollow microspheres at a scan rate of 20 mV s^–1^. b) Specific capacitances of Mn_2_O_3_ nanoparticles, single‐, double‐, triple‐ and quadruple‐shelled Mn_2_O_3_ hollow microspheres at various scan rates. c) Comparison of galvanostatic charge/discharge curves of Mn_2_O_3_ nanoparticles, single‐, double‐, triple‐ and quadruple‐shelled Mn_2_O_3_ hollow microspheres at a current density of 1 A g^–1^ in the voltage range of 0–0.45 V. d) Cyclic stability of Mn_2_O_3_ nanoparticles, single‐, double‐, triple‐ and quadruple‐shelled hollow microspheres for 2000 cycles at a current density of 1 A g^–1^.

Since the charge storage process of a supercapacitor is a surface reaction between the electrolyte ions and electrode materials,[[qv: 2b]] a detailed surface structure investigation is therefore needed to understand the mechanism behind the discrepant performance. From the TEM and SEM images we can clearly see that the shell thickness of multi‐shelled hollow microspheres is different and triple‐shelled Mn_2_O_3_ shows the thinnest shells of about 51 nm (Figure S10, Supporting Information). The Brunauer–Emmett–Teller (BET) data (Table S3, Supporting Information) show that the triple‐shelled Mn_2_O_3_ possesses the highest specific surface area of 36.55 m^2^ g^–1^ and also the largest pore volume of ≈0.518 cm^3^ g^–1^ among other multi‐shelled hollow microspheres investigated (clearly see pores on the shells in Figure S11, Supporting Information). It can be deduced that a higher specific surface area and larger pore volume could guarantee an abundance of the active sites and the easy electrolyte penetration into the materials, resulting in an improved capacitive performance. It should be mentioned that the trend of the specific capacitance changes is consistent with the changes of specific surface areas and the pore volumes of multi‐shelled Mn_2_O_3_ hollow microspheres, supporting our standpoints. However, when comes to Mn_2_O_3_ NPs, it shows a much higher specific surface area and larger pore volume, yet a lower specific capacitance. It could be explained from two aspects: first, although Mn_2_O_3_ NPs possess a larger surface area, the actual accessible reactive surface area of hollow microspheres is much larger due to the special hierarchical structure and well‐preserved interface dispersibility in the electrode layers, while the Mn_2_O_3_ NPs could lose large portion of their reactive sites due to serious accumulation and crosslink. Additionally, the unique three‐dimensional hierarchical porous multi‐shelled hollow structure can ensure the ions from both outer and inner shell to be charged and transferred, resulting in superior capacitive and rate performance.^7^

Long cycling life is also an important requirement for supercapacitors. In this regards, MnO_x_ often suffers from a limited long‐term stability because of the partial dissolution in the electrolyte during cycling, which restricts the commercial application of such low‐cost supercapacitor electrode materials.^19^ However, the construction of multi‐shelled MnO_x_ hollow microspheres is able to noticeably meliorate the cycling performance. The cycling life test of Mn_2_O_3_ NPs and hollow microspheres were examined between 0 and 0.45 V in 6 m KOH aqueous electrolyte by consecutive charge/discharge cycles at a current density of 1 A g^–1^ (Figure 4d). Interestingly, the multi‐shelled Mn_2_O_3_ hollow microspheres exhibited a much better cycling stability than the randomly aggregated Mn_2_O_3_ NPs. For instance, after 2000 consecutive cycles, the capacitance retention of the triple‐shelled Mn_2_O_3_ hollow microspheres still kept at 1517 F g^–1^ (only 7.8% loss), while Mn_2_O_3_ NPs could only retain a capacitance of 376.5 F g^–1^ with a 35.3% loss of its initial capacitance (581.7 F g^–1^). The better stability of the multi‐shelled Mn_2_O_3_ hollow microspheres can be ascribed to the superior shell structure with the shells supporting for each other, especially the exterior shell protecting the interior shells, which can undertake some mechanical deformation and swell in the redox processes and suppress the dissolution of Mn_2_O_3_ in electrolyte.

In summary, we have developed a scalable approach to fabricate multi‐shelled Mn_2_O_3_ hollow microspheres as high‐performance supercapacitor electrodes and the effect of shell structures on the capacitive performance are carefully studied. The unique architecture of these designed nanostructures allows efficient use of pseudo‐capacitive Mn_2_O_3_ nanomaterials for charge storage due to the facilitated transport of both electrolyte ions and electrons, thus rendering them with a record high specific capacitance of 1651 F g^–1^, good rate capability and remarkable cycling performance. We believe that such earth‐abundant, low‐cost, environment‐friendly, and high‐performance electrode materials achieved through a scalable and controllable process, can offer great promise in large‐scale energy storage device applications.

## Supporting information

As a service to our authors and readers, this journal provides supporting information supplied by the authors. Such materials are peer reviewed and may be re‐organized for online delivery, but are not copy‐edited or typeset. Technical support issues arising from supporting information (other than missing files) should be addressed to the authors.

Supplementary
