# Supplementary material for: pH‐Regulated Synthesis of Multi‐Shelled Manganese Oxide Hollow Microspheres as Supercapacitor Electrodes Using Carbonaceous Microspheres as Templates
Source: Adv Sci (Weinh). 2014 Dec 10;1(1):1400011. doi: 10.1002/advs.201400011 (PMC5115267; doi:10.1002/advs.201400011)
Supplement: Supplementary file 1 — Supplementary [file ADVS-1-0a-s001.pdf]

Copyright WILEY-VCH Verlag GmbH & Co. KGaA, 69469 Weinheim, Germany, 2014.

ADVANCED SCIENCE
Open Access

Supporting Information

for *Adv. Sci.*, DOI: 10.1002/advs. 201400011

pH-Regulated Synthesis of Multi-Shelled Manganese Oxide Hollow Microspheres as Supercapacitor Electrodes Using Carbonaceous Microspheres as Templates

Jiangyan Wang, Hongjie Tang, Hao Ren, Ranbo Yu, Jian Qi, Dan Mao, Huijun Zhao, and Dan Wang**

Copyright WILEY-VCH Verlag GmbH & Co. KGaA, 69469 Weinheim, Germany, 2014.

Supporting Information

pH-regulated Synthesis of Multi-shelled Manganese Oxide Hollow Microspheres for Supercapacitor Electrode Using Carbonaceous Microspheres as Templates

Jiangyan Wang,[‡] Hongjie Tang,[‡] Hao Ren, Ranbo Yu, Jian Qi, Dan Mao, Huijun Zhao, Dan Wang**

Experimental section

Synthesis of multi-shelled Mn_2O_3 hollow microspheres: All reagents (purchased from Beijing Chemical Co. Ltd.) were analytical grade and used as received. Manganese acetatetetrahydrate ($Mn(Ac)_2 \cdot 4H_2O$) was used as the metal precursor. Taking the synthesis of the quadruple-shelled Mn_2O_3 hollow microspheres as an example, a prototypical synthesis process was described as follows. First, carbonaceous microspheres (CMSs) templates were prepared through the emulsion polymerization reaction of sucrose under the hydrothermal conditions as described elsewhere.[1] Then freshly-prepared CMSs (600 mg) were dispersed in 30 mL of a 1 M manganese acetate solution (water : ethanol = 1:3, v/v) with the aid of ultrasonication. After ultrasonic dispersion for 15-30 min, the resulting suspension was stirred for 6 h at 30°C in a water bath, then filtered with a vacuum pump and washed with deionized water for three times, and then dried at 80°C for more than 12 h. Finally, the quadruple-shelled Mn_2O_3 hollow microspheres were produced after an annealing process, that is: the resultant composite microspheres were heated in a muffle furnace to 500°C in air at the rate of 1 °C min^{-1}, and kept at 500°C for 1 h, then cooled down to room temperature naturally. The shell numbers of the Mn_2O_3 hollow microspheres were tuned with the control of the solution pH values by adding hydrochloric acid into the manganese acetate solution, while the washing, drying and annealing processes remained the same. Detailed experimental parameters can be found in Table S1.

Materials characterization: Powder X-ray diffraction (XRD) patterns were recorded on a Panalytical X' Pert PRO MPD [Cu Ka radiation (λ = 1.5405 Å)], operating at 40 kV and 30 mA. Scanning electron microscopy (SEM) images were obtained by using a JSM-6700 microscope operating at 5.0 kV. TEM (transmission electron microscopy) images were carried out using Tecnai G2 20 S-TWIN at 200 kV. The nitrogen adsorption-desorption isotherms were measured on a Quantochrome Autosorb-1MP sorption analyzer under liquid nitrogen (-196°C) with prior degassing under vacuum at 200°C for more than 12 h. X-ray photon

spectroscopy (XPS) spectra were performed by an ESCALAB 250 Xi XPS system of Thermo Scientific, where the analysis chamber was 1.5×10^{-9} mbar and the X-ray spot was 500 μm. The XPS data were corrected with reference to C 1s (284.8 eV). The TGA-DTA (thermo gravimetric analysis - differential thermal analysis) data were collected through a DTA-60 (Shimadzu, Japan), annealed under air atmosphere with a heating rate of 1 °C min^{-1}.

Electrochemical measurements: The electroactive materials were mixed with carbon black and polyvinylidence difluoride (PVDF) binder at a mass ratio of 70:20:10 in a mortar with N-methyl pyrrolidinone (NMP) solvent and ground to form a slurry. This slurry was then spread and pressed on to the Ni foam current collector (diameter: 1.5 cm) and allow to dry in a vacuum oven at 120°C for 12 h (loading amount of active materials: ~0.6 mg). Electrochemical experiments were then performed at room temperature on the CHI 660C (CH Instruments, Inc) electrochemical workstation using the three electrode system in a beaker in 6 M KOH without removal of oxygen from the solution. An SCE (saturated calomel electrode) reference electrode and a platinum foil counter electrode were used for the cyclic voltammetry (CV) over a voltage range of 0 to 0.45 V at various scan rates (5 to 200 mV/s). The average specific capacitance (C_{sp}) determined from the cylic voltammometric curves was calculated according to Equation (1):

$$C_{sp} = \frac{1}{w\Delta v}\int_{y}^{x} idt \qquad (1)$$

Where i, Δv, and w are the current (mA), the voltage range of one scanning segment (V), and mass of the electrode material (mg), respectively. The specific capacitance was also calculated from the chronopotentiometric curves according to Equation (2):

$$C_{sp} = \frac{I\Delta t}{w\Delta v} \qquad (2)$$

where I is the charge/discharge current, Δt is the time for a full charge or discharge, w is the weight of the active electrode material, and $\Delta \upsilon$ is the voltage change after a full charge or discharge, respectively.

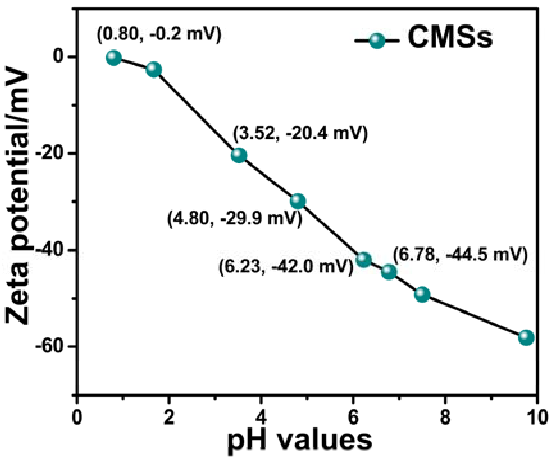

Figure S1. Zeta potentials of CMSs in aqueous solution at different pH values.

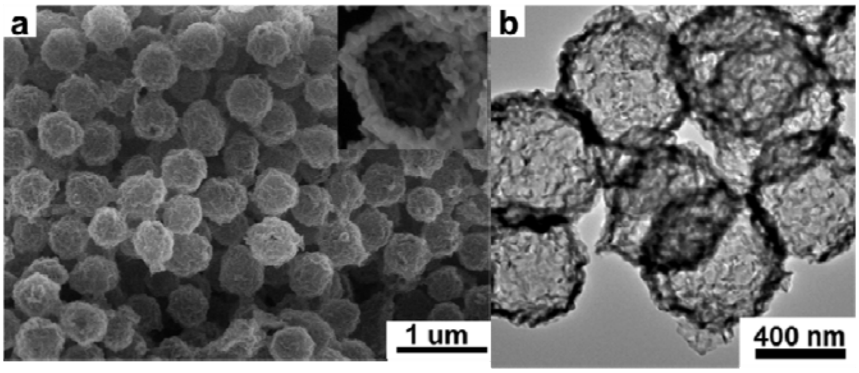

Figure S2. a) SEM and b) TEM images of the single-shelled Mn_2O_3 hollow microspheres

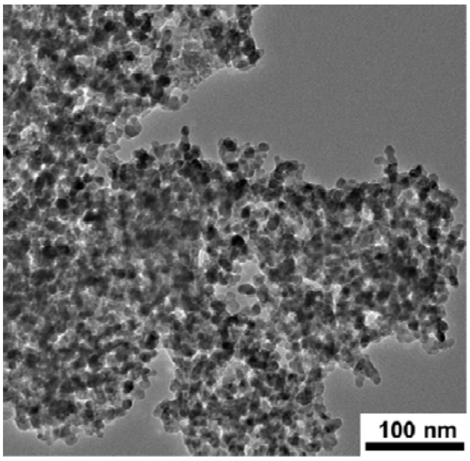

Figure S3. TEM images of as-prepared Mn_2O_3 nanoparticles

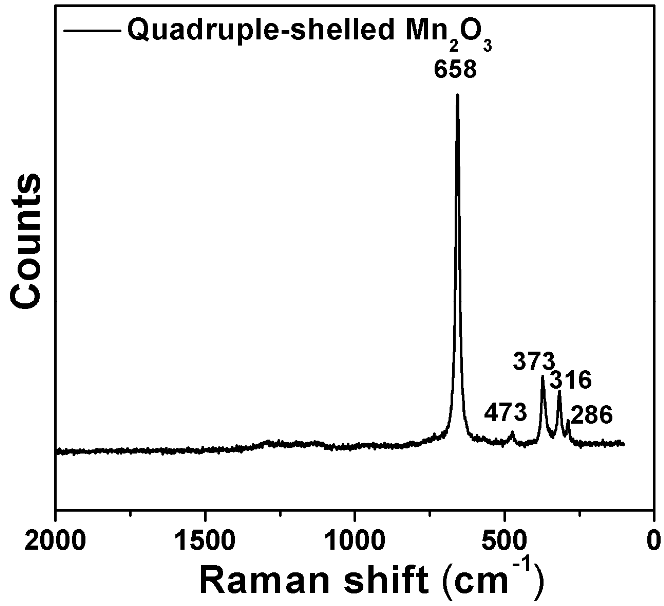

Figure S4. The Raman spectra of quadruple-shelled Mn_2O_3 microspheres.

Additionally, since Raman spectra could offer some detailed structural and compositional information, we conducted the Raman characterization to monitor the forms of carbon in multi-shelled Mn_2O_3 microspheres. As we all know, the ratio of G-band peak at about 1350 cm^{-1} and D-band peak at 1550 cm^{-1} in Raman spectra can reflect the ratio of graphitic carbon to amorphous carbon in the samples. However, in the following Raman spectra of quadruple-shelled Mn_2O_3 microspheres, we can hardly detect the existence of G- and D- band of carbon and and the Raman peaks at 658, 473, 373, 316 and 283 cm^{-1} show the characteristic crystalline vibration of Mn_2O_3 phase[1e]. Therefore, combined with our heat treatment conditions and the TGA curves (Figure S6), we can get the conclusion that there is not any kind of amorphous impurity species in the final multi-shelled microspheres.

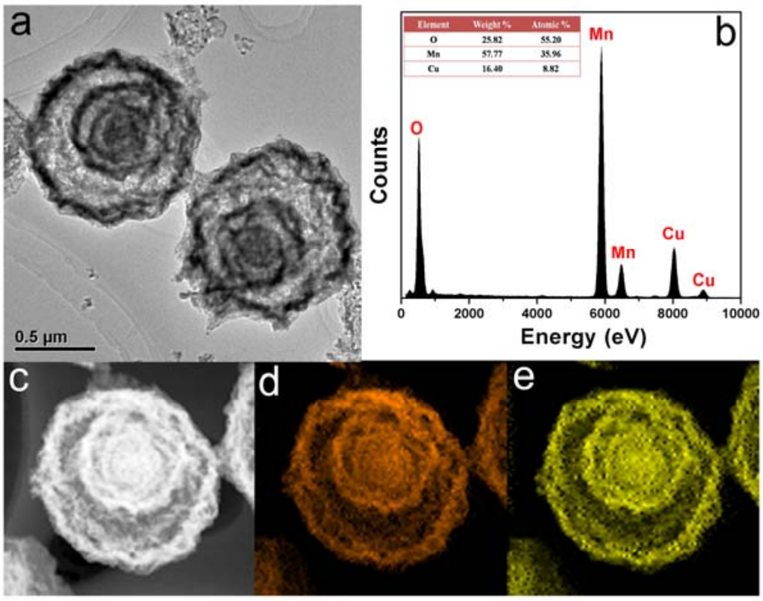

Figure S5. (a) TEM image of quadruple-shelled Mn_2O_3 microspheres, (b) EDX curve of corresponding Mn_2O_3 microspheres, the inset shows the atomic ratio of Mn, O and Cu, (c-e) HAADF-STEM element mapping images of quadruple-shelled Mn_2O_3 microspheres: (d) Mn, (e) O.

The compositional information can also be obtained by EDX analysis. As we can see from the Figure S5b, EDX curves clearly show the existence of Mn, O and Cu elements (from the cooper grid for TEM) and the ratio of Mn and O is almost 2:3, which is consistent with the compositional ratio in Mn_2O_3. Further compositional distribution is recognized by the corresponding elemental mapping (Figure S5c~5e), in which Mn and O elements are found to be uniformly distributed in the products without other impurity elements.

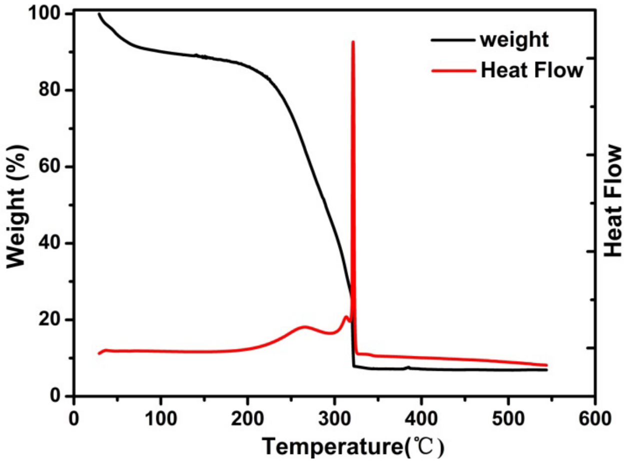

Figure S6. TGA-DTA curves of mangenese ions infused carbonaceous microspheres under air atmosphere at a heating rate of 1 °C min^{-1}.

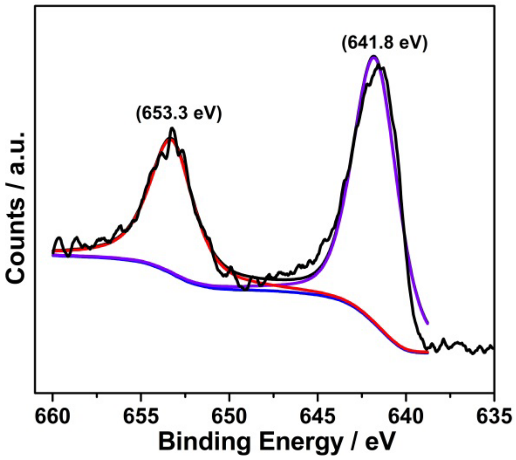

Figure S7. XPS spectra of Mn $2p_{3/2}$ and Mn $2p_{1/2}$ for triple-shelled Mn_2O_3 hollow microspheres.

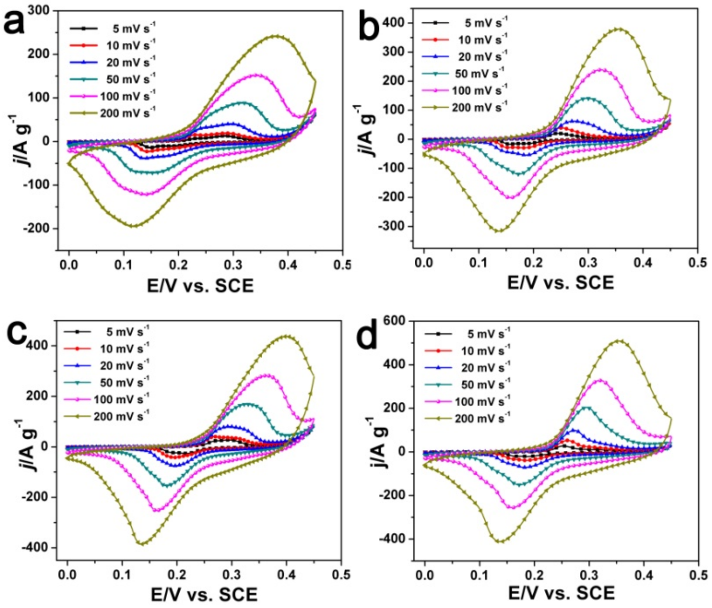

Figure S8. CV curves (a-d) of Mn_2O_3 nanoparticles, single-, double-, and quadruple-shelled Mn_2O_3 hollow microspheres in various scan rates of 5, 10, 20, 50, 100, 200 mV/s.

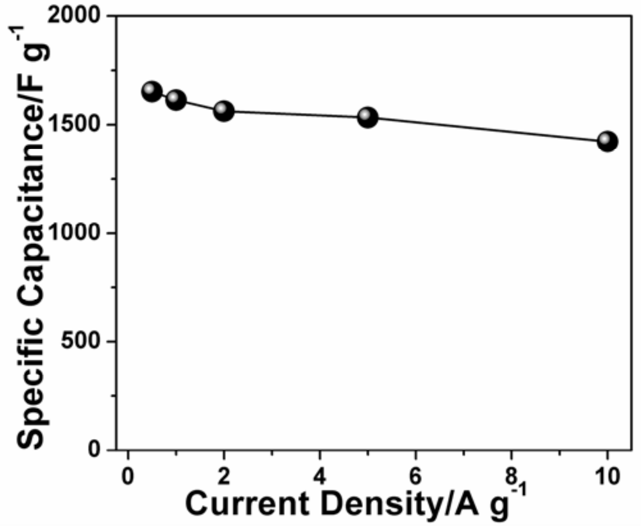

Figure S9. Specific capacitances of triple-shelled Mn_2O_3 hollow microspheres at various current densities.

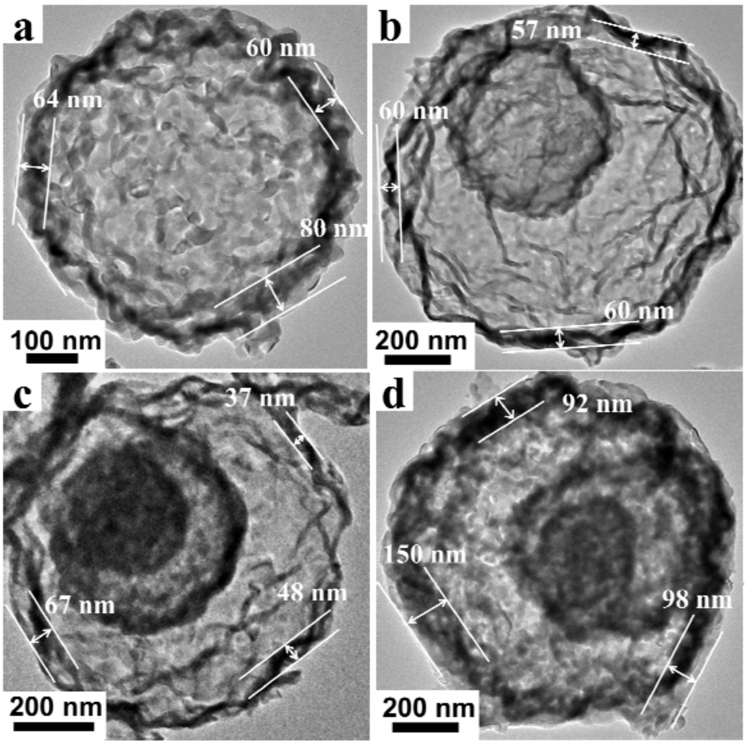

Figure S10. TEM images of single-, double-, tripe- and quadruple-shelled Mn_2O_3 hollow microspheres showing the shell thickness of 68, 59, 51 and 113 nm, repectively.

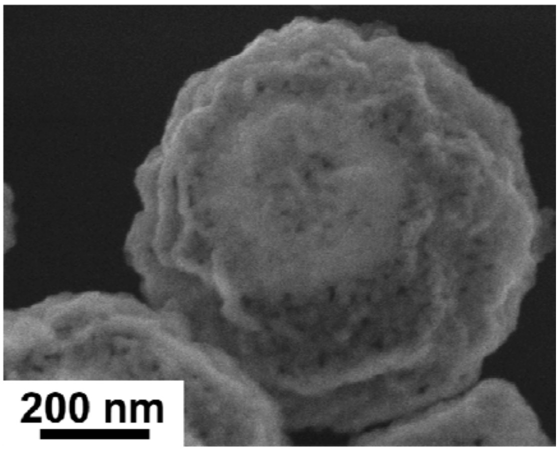

Figure S11. High resolution SEM image of the triple-shelled Mn_2O_3 hollow microspheres

Table S1. Summary of synthesis conditions of various multi-shelled Mn_2O_3 hollow microspheres and Mn_2O_3 nanoparticles

Structures	Manganese salt	Concentration (M)	Water : ethanol	Adsorption temperature (°C)	Adsorption time (h)	pH	Annealing temperature (°C)
Single-shelled	$Mn(Ac)_2{\cdot}4H_2O$	1.0	∞	24	6	3.35	500
Double-shelled	$Mn(Ac)_2{\cdot}4H_2O$	1.0	∞	24	6	4.43	500
Triple-shelled	$Mn(Ac)_2{\cdot}4H_2O$	1.0	∞	24	6	6.43	500
Quadruple-shelled	$Mn(Ac)_2{\cdot}4H_2O$	1.0	1：3	30	6	6.92	500
Nanoparticles	$Mn(Ac)_2{\cdot}4H_2O$	1.0	∞	24	6	0.79	500

Table S2. Comparison of the electrochemical capacitive performance of Mn_2O_3-based electrode materials in literatures.

Electrode material	Specific capacitance ($F\ g^{-1}$)	electrolyte	Voltage window (V)	Current load or scan rate	Reference
Mn_2O_3thin film	270 (3-electrode)	0.1 M Na_2SO_4	-0.1 - 0.9 (vs. Ag/AgCl)	1 mV/s	2
Mn_2O_3/carbon aerogel microbead	368 (3-electrode)	6 M KOH	0 - 1.0 (SCE)	1 mV/s	3
Mn_2O_3/carbon	160 (for the composite) 650 (calculated for Mn_2O_3) (3-electrode)	6 M KOH	-0.3 - 0.3 (vs. Ag/AgCl)	5 mV/s	4
rGO-Mn_2O_3/ Mn_3O_4	425 (3-electrode)	1 M NaOH	-0.2 - 0.5 (vs. Ag/AgCl)	5 mV/s	5
	133 (2-electrode)		0 - 1.4	0.2 A g^{-1}	
Mn_2O_3/ Mn_3O_4-Fe films	232.3 (3-electrode)	1 M Na_2SO_4	0 - 1.0 (SCE)	25 mV/s	6
(CNTs)/ Mn_2O_3	370 (3-electrode)	1 M Na_2SO_4	0 - 0.8	0.1 A g^{-1}	7
Mn_2O_3 nanospheres	100 (3-electrode)	6 M KOH	0 - 0.4 (SCE)	5 mV/s	8
Mn_2O_3/ Mn_3O_4 films	425 (3-electrode)	0.25 M Na_2SO_4	0 - 0.9 (SCE)	10 mV/s	9
Nanoporous MnO_x	309 (3-electrode)	1 M Na_2SO_4	0 - 0.9 (SCE)	5 mV/s	10
Triple-shelled Mn_2O_3 hollow microspheres	1651 (3-electrode)	6 M KOH	0 - 0.45 (SCE)	0.5 A g^{-1}	This work

Table S3. Specific surface area and pore volume of as-prepared multi-shelled Mn_2O_3 hollow microspheres and Mn_2O_3 nanoparticles according to the BET measurements.

Structures	Specific surface area (m^2/g)	Pore volume (cm^3/g)
Single-shelled	27.04	0.472
Double-shelled	27.71	0.505
Triple-shelled	36.55	0.518
Quadruple-shelled	30.22	0.402
Nanoparticles	117.4	0.954

References

[1] a) X. Lai, J. Li, B. A. Korgel, Z. Dong, Z. Li, F. Su, J. Du, D. Wang, *Angew. Chem. Int. Ed.* **2011**, *50*, 2738; b) Z. Dong, X. Lai , J. E. Halpert, N. Yang, L. Yi, J. Zhai, Z. Tang, L. Jiang, D. Wang, *Adv. Mater.* **2012**, *24*, 1046; c) J. Wang, N. Yang, H, Tang, Z, Dong, Q. Jin, M. Yang, D. Kisailus, H. Zhao, Z. Tang, D. Wang, *Angew. Chem. Int. Ed.* **2013**, *52*, 6417; d) S. Xu, C. Hessel, H. Ren, R. Yu, Q. Jin, M. Yang, H. Zhao, D. Wang, *Energy Environ. Sci.* **2014**, *7*, 632; e) Q. Javed, F. P. Wang, M. Y. Rafique, A. M. Toufiq, Q. S. Li, H. Mahmood, W. Khan, *Nanotechnology* **2012**, *23*, 415603.

[2] D. F. Yang, *Mater. Sci. Forum* **2012**, *706-709*, 884.

[3] X. Y. Wang, L. Liu, X. Y. Wang, L. H. Yi, C. Y. Hu, X. Y. Zhang, *Mater. Sci. Eng. B* **2011**, *176*, 1232.

[4] L. L. Zhang, T. X. Wei, W. J. Wang, X. S. Zhao, *Microporous Mesoporous Mater.* **2009**, *123*, 260.

[5] A. T. Chidemdo, S. H. Aboutalebi, K. Konstantinov, C. J. Jafta, H. K. Liu, K. I. Ozoemena, *RSC Adv.* **2014**, *4*, 886.

[6] C. C. Chen, C. Y. Yang, C. K. Lin, *Ceram. Int.* **2013**, *39,* 7831.

[7] Y. F. Zhang, M. Z. Ma, J. Yang, W. Huang, X. C. Dong, *RSC Adv.* **2014**, *4*, 8466.

[8] T. Nathan, M. Cloke, S. R. S. Prabaharan, *J. Nanomater.* **2008**, *2008*, 1.

[9] N. Nagarajan, H. Humadi, I. Zhitomirsky, *Electrochim. Acta*, **2006**, *51*, 3039.

[10] C. C. Yu, L. X. Zhang, J. L. Shi, J. J. Zhao, J. H. Gao, D. S. Yan, *Adv. Funct. Mater.* **2008**, *18*, 1544.
